# Choosing the Best Digital Health Literacy Measure for Research: Mixed Methods Study

**DOI:** 10.2196/59807

**Published:** 2025-04-08

**Authors:** Charlotte Brun Thorup, Mika Uitto, Kerryn Butler-Henderson, Sarah Wamala-Andersson, Merja Hoffrén-Mikkola, Diana Schack Thoft, Lisa Korsbakke Emtekær Hæsum, Gabriela Irrazabal, Laura Pruneda González, Katja Valkama

**Affiliations:** 1 Research Centre of Health and Applied Technology University College of Northern Denmark Aalborg Denmark; 2 Department of Radiography University College of Northern Denmark Aalborg Denmark; 3 Seinäjoki University of Applied Sciences Seinäjoki Finland; 4 School of Nursing, Paramedicine, & Healthcare Sciences Charles Sturt University Wagga Wagga Australia; 5 School of Health, Care and Social Welfare, Health and Welfare Mälardalen University Västerås Sweden; 6 Department of Nursing University Collage of Northern Denmark Aalborg Denmark; 7 RMIT University, AU Barcelona Spain; 8 Health Research Institute of the Principality of Asturias (ISPA), Spain Asturias Spain

**Keywords:** digital health literacy, digital literacy, Horizon Europe, EU, health technology, life expectancy, health literacy, chronic disease, digitalization, digital health service, digital health intervention, technology, healthcare

## Abstract

**Background:**

The global demographic shift towards longer life expectancy and complex health needs is increasing the number of people with chronic diseases, placing pressure on health and care systems. With the digitalization of healthcare, digital Health Literacy (dHL), or the use of digital skills in health, is gaining importance. It involves navigating digital health information, using digital tools effectively, and making informed health decisions. Measuring dHL can help identify gaps and develop strategies to improve dHL and health, ensuring citizens equal opportunity to participate in a digital healthcare system. The European project “The Improving Digital Empowerment for Active and Healthy Living (IDEAHL)” with the objective to empower European Union citizens to use digital instruments to take a more active role in managing their health and well-being creates the base for this overview

**Objective:**

This paper aims to conduct an overview of existing assessment tools for measuring dHL and recommend strategies for choosing relevant assessment tools.

**Methods:**

This study was carried out as a mixed method study initiated by a scoping review (10 scientific databases, 14 databases with grey literature and 14 predefined reports) in addition to three papers published after finalisations the literature search in IDEAHL, followed by a qualitative workshop study and a final analysis combining results.

**Results:**

The literature search resulted in 33 papers on dHL instruments, that was analyzed together with three recently published reviews and findings from a workshop with 13 champions (understood as professionals with expertise in HL and dHL) from five countries (Spain, Denmark, Sweden, Australia, and Germany) representing the health sector or health literacy research. Future tools should adapt to the latest trends and technologies, considering attitudes towards digital health and trust in its services. They should identify beneficiaries of digital health services, measure the impact of dHL interventions, and objectively evaluate functional skills. These tools should be evidence-based, validate instruments, interpret dHL results, and capture diverse experiences to reveal health behaviour changes.

**Conclusions:**

The eHealth Literacy Scale (eHEALS), despite being the most frequently utilized tool, has limitations in scope and adaptability. Future tools need to reflect digital trends, encompassing individual skills. However, it is important to note that the ‘adequacy’ of dHL is context-specific and relies on healthcare systems and the technology provided, particularly the user interface. The focus should be on health improvement, not just elevating dHL levels. A comprehensive approach to dHL assessments addressing diversity and relevance is crucial. Ethical considerations in dHL, including privacy and data security, are important due to potential feelings of shame among those with low literacy levels.

## Introduction

The demographic profile is changing worldwide, with longer life expectancy and increasingly complex health needs, leading to a rise in the number of people living with chronic diseases and placing growing pressure on the health and care system [1]. The challenge of meeting the needs of this expanding population will fall upon an already overwhelmed system struggling to cope with both current and evolving demands [2]. As a result, the role of modern consumers is shifting from paternalistic models of health care, in which patients are passive recipients, to a model that encourages more active citizen involvement in their own health and care [3]. However, this transition requires a certain level of personal empowerment and self-management, 2 concepts in which health literacy (HL) plays a crucial role [4]. HL is an emerging area of research that focuses on individuals’ ability to make informed health choices that maintain and promote well-being, based on their capacity to access, evaluate, and use health-related information [5]. According to the World Health Organization [6], HL represents the personal knowledge and competencies accumulated through daily activities, social interactions, and across generations. HL encompasses more than the ability to access websites, read pamphlets, and follow prescribed health-seeking behaviors. It includes the capacity to critically evaluate health information and resources, as well as the ability to communicate and express personal and societal needs for promoting health. A high level of HL enables individuals to understand medication labels, test results, and health care instructions [6].

The increasing role of technology in health care is leading to the digitalization of the health care system, and the concept of digital HL (dHL) is gaining more attention. In short, dHL refers to the use of digital skills in health. It involves the knowledge and abilities required to navigate the digital landscape of health information, utilize digital tools and resources effectively, and make informed decisions about one’s health [6]. Digitally health-literate individuals can use electronic sources such as computers, the internet, and social media to find and apply health information and services [7]. Those with high dHL can efficiently access health information through websites, health apps, and other digital platforms. However, individuals with limited dHL may struggle to navigate these online resources [7]. A high level of dHL enhances self-management by enabling the use of digital tools to track health metrics, schedule appointments, understand one’s digital health record, and monitor chronic conditions via telemonitoring and health apps [7].

Both HL and dHL require the ability to critically assess the reliability and credibility of health information sources. HL enables individuals to evaluate the trustworthiness of printed materials, while dHL extends this skill to digital sources, such as health websites and social media [7].

While it is important to consider dHL in terms of an individual’s skills and abilities, it should also be understood in a broader, relational context. The “adequacy” of an individual’s (d)HL is context specific and depends on the demands and complexities of health care systems and health information sources [8]. For instance, if a digital information source or service is accessible and uses clear language, even lower levels of dHL may be sufficient. Additionally, the measurement of HL and dHL varies depending on the definition, methodology, time frame of studies, country, and target population.

Measuring dHL helps identify gaps and develop strategies to improve them, ensuring everyone has an equal opportunity to understand and participate in a digital health care system. Clinical screening tests can identify individual patients’ difficulties in understanding and using health information. However, these measures have been criticized for being time-consuming in clinical settings [9], failing to fully cover HL concepts, and potentially causing shame and stigma among individuals with low literacy levels [8,10]. These tests are typically administered face-to-face. By contrast, measuring HL at the population level can help identify vulnerable groups, design targeted initiatives, address inequalities, and evaluate the success of interventions [8].

The Improving Digital Empowerment for Active and Healthy Living (IDEAHL) initiative [11] aims to empower European Union (EU) citizens to use digital tools to take a more active role in managing their health and well-being. The IDEAHL Consortium, composed of 14 multidisciplinary partners from 10 EU Member States, acts as an enabler by raising awareness among citizens and professionals about the potential of digital tools for active and healthy living. This awareness is fundamental to successfully using digital health technologies and improving both HL and dHL, ultimately leading to better health outcomes. IDEAHL has analyzed various approaches to monitoring and assessing HL and dHL levels in the EU through a comprehensive scoping review. It aims to identify the most suitable instrument for each context based on lessons learned from best practices and “Champions.” In IDEAHL, Champions are defined as professionals or individuals from services, organizations, municipalities, or regions with expertise in HL and dHL who have successfully implemented initiatives or interventions related to HL or dHL [11].

This paper aims to provide an overview of existing assessment tools for measuring dHL and recommend strategies for selecting relevant tools.

As the field of dHL assessment tools continues to develop and health care systems become increasingly digital, this overview focuses specifically on dHL, excluding assessment tools that solely measure HL.

This overview aims to address the following research questions:

- What assessment tools are used to measure dHL?
- How do Champions reflect on selected studies of dHL and strategies for choosing relevant assessment tools?

To answer the first research question, we present relevant findings from the IDEAHL scoping review, which examines dHL assessment tools used in recent research within the EU. To broaden the scope, 3 recently published review studies conducted outside the EU were included after the completion of the IDEAHL scoping review.

To answer the second research question, we conducted a qualitative workshop study with professionals specializing in HL and dHL (Champions) to assess their experiences, perspectives, and opinions on using these assessment tools.

## Methods

### Study Design

Inspired by an explanatory sequential design, the study used a mixed methods approach. The first step involved a scoping review supplemented by 3 additional papers, followed by a qualitative study and a final analysis integrating both studies.

### Scoping Review: What Assessment Tools Are Used to Measure dHL?

Inspired by the Joanna Briggs Institute [12], a scoping review protocol was developed and followed (available upon request from the first author [CBT]).

From June to November 2022, the scoping review was conducted as part of the IDEAHL project, aiming to review existing monitoring mechanisms and synthesize data to assess HL and dHL levels in the EU. The search period covered studies published between January 2018 and May 2022. The scoping review addressed the following research subquestions relevant to this paper: (1) What monitoring and assessment tools, methods, and indicators exist for measuring HL and dHL in the EU? (2) How are validation and sensitivity documented for these monitoring and assessment tools, methods, and indicators? The search concept and context are presented in Textbox 1.

**Concept and context for the scoping review.**

**1. Concept**

- Literature related to the definition of health literacy (HL) and digital health literacy (dHL) as in the search protocol.
- Include the terms HL and dHL (or the equivalent in the national language).
- Definition of dHL:
  It involves the skills and knowledge needed to navigate the digital landscape of health information, use digital tools and resources effectively, and make informed decisions about one’s health [6].
- Monitoring and evaluation of HL and dHL indicators, tools, methods, and frameworks.
- Levels of HL and dHL among population groups.

**2. Context**

- Individual, local, regional, and national initiatives.
- Public and private initiatives and services within the health area.

### Search Strategy

To answer the research questions several inclusion and exclusion criteria were set (Table 1).

To ensure a comprehensive search, several scientific databases were consulted, including sources containing gray literature (Textbox 2). Additionally, relevant literature already known to the Consortium partners was included, as these sources had contributed to establishing the need for IDEAHL.

A search strategy was developed for each scientific database (Textbox 2) using keywords from titles, abstracts, and index terms. For example, the strategy for PubMed—presented in Multimedia Appendix 1—served as the primary basis for constructing the search string, with several test searches conducted. Similar search strategies were applied to all included databases. Keywords included variations of HL, dHL, eHealth literacy (eHL), and related terms, along with a range of keywords representing monitoring, assessment, and validation of dHL tools. The inclusion criteria required studies to either measure dHL levels or validate dHL assessment tools.

Both qualitative and quantitative papers were included in the study. No critical appraisal of individual sources was conducted, as gray literature was also included, which falls outside the scope of traditional appraisal tools [12]. However, papers from scientific databases had already undergone peer review, indicating a certain level of critical appraisal.

**Table 1:** Inclusion and exclusion criteria for the scoping review.

Criteria	Inclusion criteria	Exclusion criteria
Date	From January 2018 to May 2022	All other years
Study design	Any kind of studies	Published comments, editorials, letters, and study protocols
Countries	European Union countries: Austria, Belgium, Bulgaria, Croatia, Cyprus, Czechia, Denmark, Estonia, Finland, France, Germany, Greece, Hungary, Ireland, Italy, Latvia, Lithuania, Luxembourg, Malta, Netherlands, Poland, Portugal, Romania, Slovakia, Slovenia, Spain, and Sweden	All other countries
Languages	English, Danish, Finnish, Norwegian, Swedish, German, French, Italian, Portuguese, and Spanish.	All other languages
Population	Any populations	N/A^a^

^a^N/A: not applicable.

**List of databases searched and additional sources.**

**1. Scientific databases**

AMED, Scopus, Web of Science, APA PsycINFO, CINAHL Complete, Cochrane Library, MEDLINE, PubMed, Embase, ERIC

**2. Gray literature**

International THA Database, NICE, Google Incognito, Google Scholar, Mednar, OpenDOAR, Open Access, DART Europe, ClinicalTrials.gov, WHO data collection and clinical trials, Cordis and EU trials register, JMIR Proceedings, OAIster, and Bielefeld Academic Search Engine

**3. Additional sources**

HL Atlas [13], HL Europe [14], Policy Précis by EuroHealthNet [15], eHealth Action Plan 2012-2020 [16], Horizon 2020 [17], IC-Health [18], Digital Health Europe [19], HL in the Nordic Countries [20], DHE’s practice catalogue [21], European HL Survey [22], Health Literacy Tool Shed [23], The HLS-EU questionnaire [24], The M-POHL network action [25], WHO HL Road Map [26]

### Study Selection (Screening Process)

The systematic review tool Covidence was used to manage the review process [27]. First, references retrieved from all searches were uploaded to the software, where duplicates were automatically removed. Next, titles and abstracts were screened for eligibility. Two reviewers independently screened the titles and abstracts using the predefined inclusion criteria, with a third reviewer resolving any disagreements. Following the initial screening, the full texts of the selected studies were reviewed using the same procedure. Before the data extraction phase, a second round of full-text quality review was conducted by partners with the most research experience.

### Data Extraction

The data charting and extraction process was guided by the research questions, resulting in the collection of information on the author, year of publication, target group, country, dHL tool used, dHL level measured, and any comments on the validation of the assessment tool. Specifically, the extraction focused on whether the tool was validated in the article itself, in a previous research article, or if no mention of validation was made.

The characteristics of each extracted study were summarized in tables at the country level and supplemented by a narrative presentation. Descriptive statistics were used to report the prevalence of study characteristics.

After the completion of the scoping review in IDEAHL, the authors identified 3 relevant review studies on dHL instruments published in countries outside the EU. The results from these additional studies are presented narratively. Including these papers was considered important, as they represent the latest knowledge on dHL instruments. Although they were not part of the IDEAHL review due to falling outside its inclusion criteria—being conducted in non–EU countries—they still provide valuable insights.

### Workshop: How Do Champions Reflect on Selected Headings of dHL and Strategies for Choosing Relevant Assessment Tools?

The scoping review served as the foundation for the themes discussed in the workshop with individuals identified as Champions (also referred to as knowledge users). The workshop aimed to gather qualitative insights into the Champions’ experiences, perspectives, and opinions on using various instruments to measure or assess HL and dHL [11]. Participants were also asked to identify key obstacles, challenges, and areas for improvement in assessment tools for HL and dHL. In this overview, only findings related to dHL are presented.

### Participants

The Champions in IDEAHL were defined as professionals or individuals from services, organizations, municipalities, regions, etc, who had successfully implemented initiatives or interventions related to HL or dHL [11,28].

### Recruitment of Champions

Champions were identified during the scoping review process as authors of papers in which HL and dHL interventions were categorized as “best practice” due to their success in improving 1 or more HL- or dHL-related outcomes. Contact details were retrieved from the papers identified in the scoping review or from online sources. Additional Champions were identified through the networks of HL and dHL specialists linked to the IDEAHL Consortium partners [28]. The partners in the IDEAHL Consortium were asked to identify potential national Champions who had conducted successful initiatives or interventions related to HL or dHL and to invite them to the workshops. This included experts from both Europe and beyond. The inclusion criteria for successful initiatives or interventions required that Champions had demonstrated an increase in HL or dHL levels within their target groups or had contributed to improved health outcomes. A formal invitation and agenda were sent in January 2023 and circulated by the partners to a broad group of potential Champions. Additionally, the invitation was shared on social media to engage all key players in the field in a collaborative dialogue.

### Setting

The workshop was held online via Microsoft Teams (Microsoft Corporation) to accommodate Champions from various locations worldwide [28]. Based on the findings from the scoping review, a structured plan for the workshop discussions was developed. First, the IDEAHL project was introduced, followed by a presentation of the scoping review results. Participants then discussed indicators for measuring HL and dHL, as well as appropriate assessment tools. This approach was inspired by the Joanna Briggs Institute guideline on engaging knowledge users (Champions) in scoping reviews [29]. Accordingly, the Champions were invited to share their own experiences to validate the findings and assess their implications, ensuring they were relevant and meaningful to the communities [29].

To ensure full engagement with participants, the workshop was conducted by 5 academic partners with experience in qualitative research (KV, MU, MHM, CBT, and DST), along with 1 research assistant. The researchers alternated between presenting the review findings, posing discussion questions, and providing additional comments or follow-up questions as needed. The research assistant managed the technical aspects of Microsoft Teams, took notes, and contributed by suggesting comments or questions for the researchers to address.

The workshop was recorded and transcribed, with additional notes taken by 1 researcher to supplement the transcript. All qualitative data (notes and transcripts) were analyzed using deductive content analysis [30]. The analysis followed these steps: (1) defining key aspects to examine by developing a categorization matrix, (2) thoroughly reading the text multiple times, (3) coding the text based on the matrix, and (4) identifying themes through analysis and abstraction using the matrix [30].

### Ethics Considerations

The following ethical considerations were taken into account regarding the involvement of the Champions. Participation was voluntary, and only names, email addresses, and organizational affiliations were collected via Webropol (Webropol Oy). The Webropol form included a combined data information document in accordance with the EU General Data Protection Regulation (2016/679), Articles 13 and 14. The recording and notes were stored in the Microsoft Teams group created for IDEAHL. Informed consent was obtained verbally by asking participants to agree to the meeting being recorded and used for publication within the IDEAHL project. The IDEAHL project followed an established ethics protocol, and each participating country obtained ethical approval according to its national regulations, if required.

## Results

### Findings From the Scoping Reviews

**Figure 1:**
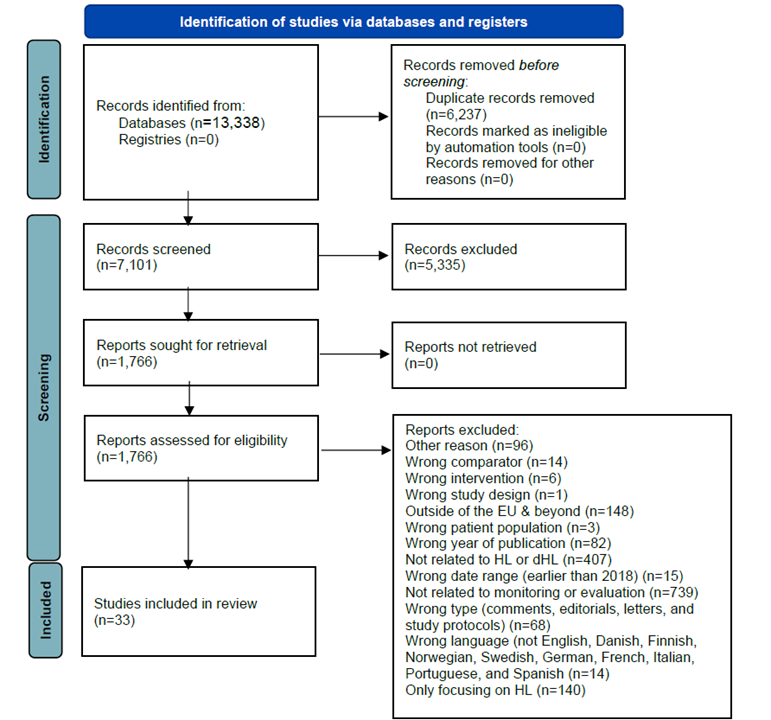
Prisma diagram over scoping review from IDEAHL.

The scoping review identified 33 papers on dHL published in the EU between 2018 and 2022. These papers focused either specifically on dHL or in combination with other HL measures [31]. Among the 7101 papers screened based on their titles, 2153 were identified as conflicting (Figure 1 and Multimedia Appendix 2).

The scoping review indicated a growing focus on dHL, with 44% of EU countries having published at least one article on dHL levels or the validation of dHL assessment tools [31]. However, the number of available tools for measuring dHL remains limited, and their scope is restricted in terms of what they assess [31].

The scoping review identified 10 different dHL measurement tools, including the eHealth Literacy Scale (eHEALS), eHealth Literacy Assessment (eHLA), eHealth Literacy Questionnaire (eHLQ), Digital Health Literacy Instrument (DHLI), eHEALS-Carer, the digital health literacy module of the Health Literacy Survey 2019 (HLS19-DIGI), and 4 adaptations of DHLI related to the COVID-19 pandemic (Table 2). Most of these instruments had only been used once, with eHEALS being the most frequently utilized, appearing in 18 EU studies and translated into most languages. The included studies targeted diverse populations, with sample sizes varying significantly (Table 2). Most studies indicated that the dHL assessment tool had been validated in previous research, validated the tool itself, or validated a translated version within the study (see Multimedia Appendix 3 for a more detailed description).

The data are derived from the IDEAHL scoping review [31], with a more detailed description of the 33 papers available in Multimedia Appendix 3. Notably, only 2 of the articles focused on dHL in children or adolescents, highlighting a gap in research on younger populations in Europe. Based on the scoping review findings, no single assessment tool could be universally recommended across European countries and diverse target populations.

**Table 2:** Different dHL^a^ tools used in the scientific literature (n=33 articles) in European Union countries with different target groups between 2018 and 2022 [31].

Assessment tool (frequency of use)	Country (target group)	Sample size or range of sample sizes (people)
eHEALS^b^ (n=18)	Austria (children and adolescents), Germany (general population, n=2; patients, n=3; carers of people with illnesses), Greece (university students and health care workforce), Hungary (general population), Ireland (carer of people with illnesses and health care workforce), Italy (university students and older adults), Poland (general populations, n=2), Sweden (general populations, n=2), and migrants (n=2)	14-1527
eHealth Literacy Assessment (n=4)	Denmark (university students, general population, and patients) and Germany (health care workforce)	113-475
eHealth Literacy Questionnaire (n=3)	Denmark (general population, patients, and health care workforce)	194-475
DHLI^c^ (n=2)	Denmark (university students) and Germany (children and adolescents)	490-1518
eHEALS-Carer (n=1)	Cyprus (carers of people with illnesses) and Greece (carers of people with illnesses)	101
HLS19-DIGI^d^ (n=1)	Portugal (general population)	1247
Five aspects of DHLI adapted to the context of the COVID-19 pandemic (n=1)	Germany (university students)	14,916
COVID-19 DHLI (n=1)	Italy (university students)	3025
DHLI adapted to the COVID-19 pandemic (n=1)	Portugal (university students)	1815
Three subscales of DHLI adapted for COVID-19 (n=1)	Slovenia (university students)	3621

^a^dHL: digital health literacy.

^b^eHEALS: eHealth Literacy Scale.

^c^DHLI: Digital Health Literacy Instrument.

^d^HLS19-DIGI: the digital health literacy module of the Health Literacy Survey 2019.

### Findings From Recent Reviews on dHL Beyond the EU Countries

Recently, 3 systematic literature reviews have examined dHL assessment tools and their application. Faux-Nightingale et al [32] analyzed 53 papers focused on adults and 3 on adolescents, identifying the use of dHL tools such as DHLI, eHEALS, eHLA, and Technology-Enabled Health Literacy Instrument (TeHLI), with eHEALS being the most frequently used. These questionnaires primarily utilized ordinal-based scoring methods, mostly Likert scales, to assess various eHealth-related domains. For children and adolescents, surveys were the predominant method for gathering information, with the eHEALS questionnaire consistently applied across all 3 studies. The authors suggested that future questionnaires should incorporate additional factors, such as attitudes toward digital health care provision, social influences, and the usability of digital health resources, which may impact an individual’s willingness to engage with them [32]. They further concluded that future research would benefit from the development of an objective questionnaire or platform that assesses functional skills [32]. Additionally, they noted that eHEALS does not adequately measure dHL in a way that allows for identifying a population’s ability to engage with digital health resources [32].

Délétroz et al [33] examined 42 articles and reported the use of several dHL assessment tools, including TeHLI, eHLQ, eHLA, DHLI, HLSI, HLS19-DIGI, and eHEALS (including eHEALS-Extended), with the latter 2 being the most extensively studied. The study assessed the content validity of these instruments, finding that eHEALS had moderate-quality evidence for comprehensibility, but inconsistent low-quality evidence for relevance and insufficient very low-quality evidence for comprehensiveness. Across the studies reviewed, there was insufficient evidence regarding the psychometric qualities of any of the tools. The study also attempted to identify patient-reported outcome measures related to eHL in adults. However, due to limited available evidence (n=6), no definitive conclusions were drawn. The authors suggested that dHL assessment tools should not only evaluate individual knowledge and skills but also consider perceptions of the health care system, including trust in its ability to manage one’s condition [33]. On an individual level and in daily clinical practice, dHL tools should help determine the extent to which a patient can benefit from eHealth tools and interventions based on their dHL level [33].

On a population level, a well-designed measurement instrument could help identify vulnerable subgroups that face additional challenges in accessing health care due to digitalization.

Despite the limited evidence on whether improving patients’ dHL leads to better health outcomes, clinical practice should consider whether the tool can effectively measure the impact of eHealth interventions [33]. Furthermore, the application of dHL tools must align with both individual and organizational needs. In clinical settings, these tools should support health care professionals in making informed decisions about a patient’s ability to benefit from eHealth tools, as suggested by Délétroz et al [33].

Lastly, Lee et al [34] analyzed 42 articles and examined 7 dHL instruments: eHEALS (including eHEALS-Extended), DHLI, eHLA, eHLQ, and TeHLI. Regarding the theoretical and conceptual frameworks underpinning these instruments, eHEALS and TeHLI are based on the Lily model and self-efficacy theory, as well as the transactional model of eHL, respectively, while eHLA and eHLQ were developed using the eHL Framework. The authors highlighted that the rapid advancement of digital and technological developments necessitates an updated dHL tool that reflects the evolving attributes and skills required for the social aspects of eHealth in today’s digital environment [34]. Additionally, they emphasize that such a tool should assess individuals’ ability to access online health care services and the internet in general. Furthermore, they suggest that dHL instruments should be tailored to encompass the attributes and skills necessary for navigating the social nature of eHealth and the broader digital landscape [34].

### Findings From Workshop With Champions

The workshop was held on January 27, 2023, and lasted for 3 hours. A total of 41 participants registered, including 22 identified as Champions. Of these, 13 attended the online workshop. Table 3 provides an overview of the nationalities and professional roles of the participating Champions.

Although this overview focuses on dHL, some reflections on HL tools are relevant when discussing dHL tools, as the 2 are interrelated. Therefore, the findings incorporate both HL and dHL perspectives.

**Table 3:** Basic characteristics of the participants in the workshop.

Characteristics		Participants, n
**Country**		
	Spain	7
	Denmark	3
	Sweden	1
	Germany	1
	Australia	1
**Professional role**		
	Health literacy researcher	6
	Patient organization professional	2
	Health and food safety professional	1
	Educational development professional	1
	Mental health literacy program coordinator	1
	No information available	2

### Different dHL Instruments Suit Different Settings and Target Groups

In the online workshop, the Champions were presented with the scoping review findings, followed by in-depth discussions. They highlighted that it is not objectively possible to recommend a single measurement instrument for assessing the dHL levels of EU citizens. Instead, they emphasized that different instruments are suited to different settings and target groups. The choice of an assessment tool should always align with the target group, the purpose of the measurement, and the specific context. Additionally, the popularity of a dHL instrument in scientific literature does not necessarily indicate that it is the most suitable option.

Evaluation is a good idea, like Realistic evaluation, conducting a program theory, what works for whom, under which circumstances, and why? By that way of researching or evaluating, you cannot only see what works, but also why it works. I think that is crucial question to address regarding this

determination of persons or groups of persons dHL

### A Multidimensional dHL Instrument Is Needed

According to the Champions, the best dHL instrument is one that aligns with the purpose of the measurement and is supported by a solid evidence base. They also emphasized that an appropriate measurement instrument should capture the multidimensional nature of dHL and include qualitative components and questions that assess the health outcomes of interventions aimed at improving dHL.

Qualitative part would be more like, how do you experience that you have been affected, or in what have you learned...we have asked very open questions, like what did you benefit from. What have you done. Not only what you have learned, but also has this affected you in any way, have you changed something in the way you work on your health.

This is not the case for some of the earliest instruments that remain popular in research, as their validation was conducted a long time ago.

### The Choice of dHL Instruments Is Influenced by the Available Resources

Furthermore, the Champions highlighted that the choice of an assessment tool is sometimes influenced by the availability of the instrument in the required language. Because of limited resources, researchers may opt for an existing instrument rather than undertaking the complex process of translating and validating a newer, potentially more suitable measurement tool.

### Specific Needs May Drive the Development of Self-Created dHL Instruments

Moreover, the Champions pointed out that individual research interests might lead some researchers to develop their own measurement instruments to address specific needs rather than selecting from existing options.

The validation of self-created instruments was not discussed. However, the Champions emphasized that future assessment instruments should have a strong evidence base and be appropriate for their intended purpose. This perspective challenges the feasibility of identifying a single, universally applicable assessment instrument.

The Champions emphasized the importance of considering the entire development process of dHL instruments, including how they are created and who is involved. They suggested that validation should go beyond instrument validation to include the validation of how the results of dHL measurements are interpreted.

You will hear me talk about validity, not in terms of the instrument. Validity theory is that it is not the instrument, but the interpretations that derive from the data. And whether the interpretations are valid for the measurement purpose and decisions that needs to be made from those.

Careful attention should be given to the interpretations drawn from data, as it is easy to reach incorrect conclusions. Continuous assessment is necessary to ensure that interpretations remain accurate and valid.

To summarize, the scoping review, the 3 supplementary papers, and the workshop with Champions indicate that no single measurement instrument can be objectively recommended to assess dHL levels across EU citizens. Future assessment tools need to be updated and adapted to emerging trends, technologies, and digital health services. These tools should capture attitudes and perceptions toward digital health provision, trust in digital health services, and the social aspects of digital health engagement. Moreover, dHL tools should identify who might benefit from specific digital health services and assess the extent to which interventions aimed at increasing dHL improve health, self-management, or other health-related outcomes. They should also objectively evaluate functional skills and incorporate user interface considerations, as usability plays a crucial role in individuals’ willingness to engage with digital health services. Additionally, future tools should be evidence based, incorporating both instrument validation and validation of result interpretations. They should be multidimensional, allowing researchers and practitioners to determine what works, for whom, under which circumstances, and why. Including qualitative components can help uncover experienced benefits and changes in health behavior. Finally, there remains a notable research gap in dHL, particularly concerning younger populations, highlighting the need for further investigation in this area.

## Discussion

### Principal Findings

This paper aimed to provide an overview of existing assessment tools for measuring dHL and to recommend strategies for selecting appropriate tools. It focused on identifying the most relevant dHL assessment tools and exploring how Champions reflect on selected dHL studies.

Future dHL assessment tools must adapt to emerging trends and technologies. They should consider factors such as general attitudes toward health, digital health, trust in digital health services, and social aspects. These tools should identify individuals who could benefit from specific digital health services and assess the impact of dHL interventions on health-related outcomes. Additionally, they should objectively evaluate functional skills, account for user interface aspects, and be grounded in strong evidence. Validation should encompass both the instrument itself and the interpretation of dHL assessment results. Furthermore, the tools should be multidimensional, capturing diverse experiences and revealing the benefits and behavioral changes associated with improved dHL.

### Interpretation of Results

According to the scoping review, the eHEALS tool was the most frequently used. However, its limitations have become increasingly apparent in the rapidly evolving digital landscape. eHEALS has been criticized for measuring a narrow scope of eHL and is considered outdated, as it was developed in 2006—before the widespread adoption of advanced technologies, online interactions, and social media in daily life and health care (Web 2.0) [34,35]. The Champions also raised similar concerns, highlighting that this issue is not exclusive to eHEALS. They emphasized the need for future assessment tools to be continuously updated and adapted to reflect emerging trends, technologies, and digital health services. Nguyen et al [36] state that the history of (d)HL tools has evolved since the 1970s, highlighting the need for careful selection or development of tools that align with the contemporary understanding of dHL. The evolution of (d)HL assessment tools has been dynamic, requiring adaptability, responsiveness to technological shifts, and a strong commitment to measurement precision [36]. Balancing technological advancements with the validity and reliability of dHL assessment tools remains a significant challenge for researchers and practitioners. Furthermore, as a self-reported subjective scale, eHEALS lacks objective testing to validate participants’ self-assessments [32]. Its developer, Norman [37], has acknowledged this limitation and suggested that incorporating an interactive subscale could improve the tool by enabling a more objective, performance-based assessment.

In line with the perspectives shared by the Champions, the review studies highlight that dHL tools should assess not only individual knowledge and skills but also individuals’ perceptions of the health care system, their trust in its ability to manage their conditions, and their capacity to access digital health care services. This expanded definition, which incorporates social and personal dimensions, emphasizes the need for dHL tools to account for users’ diverse capabilities, experiences, and expectations within the digital health care landscape. Such assessments must go beyond mere technical proficiency, prompting a reevaluation of the multidimensional nature of dHL [33,34].

The Champions emphasized that interventions targeting dHL in health care settings should ultimately aim to improve health outcomes, rather than focusing solely on increasing dHL levels in individuals and organizations.

Fujioka et al [38] conducted a comprehensive scoping review of reviews to understand the challenges associated with virtual care accessibility among underserved populations. They identified 6 key thematic areas: (1) the individual’s orientation toward health-related needs, (2) the individual’s orientation toward health-related technology, (3) the individual’s digital literacy, (4) technology design, (5) health system structure and organization, and (6) social and structural determinants of access to technology-enabled care. This suggests that an ideal dHL assessment tool should capture all these dimensions rather than focusing solely on dHL itself [38].

Balancing objective and subjective aspects of dHL assessment, as recommended by Crocker et al [39], enhances the utility of these tools by ensuring a comprehensive evaluation of individuals’ actual abilities and perceptions in navigating digital health. The success of dHL tools depends on their technical accuracy, practicality, and usability, requiring consideration of their real-world applicability in clinical settings, including potential time constraints and equipment needs [39]. Similarly, Kickbusch et al [8] emphasized that the adequacy of an individual’s dHL is context specific, influenced by health care systems and the clarity and accessibility of digital information sources. If digital sources are well-designed and accessible, lower dHL levels may still be sufficient. As dHL is not a static competency but a fluid one that evolves with the digital landscape, assessment tools must be adaptable to changing technologies, health care structures, and user needs. The measurement of dHL is also affected by variations in definitions, methodologies, time frames, countries, and target populations, further underscoring the importance of flexible and multidimensional assessment approaches.

This comprehensive approach ensures that dHL tools remain relevant, up-to-date, and capable of addressing the diverse dimensions of dHL, thereby contributing to a more holistic understanding of its impact on health care outcomes. At a broader level, dHL tools have the potential to identify vulnerable subgroups facing challenges in the digitized health care landscape, offering valuable insights into digital health disparities at the population level [39]. Moreover, the intersection of gender, socioeconomic status, cultural background, and educational level with dHL requires a comprehensive approach. Effective dHL tools should integrate these intersecting factors to enhance inclusivity and relevance across diverse populations, acknowledging that individuals’ abilities to engage with digital health resources are shaped by these contextual elements. By addressing these complexities, dHL assessments can provide a more nuanced understanding of digital health access and literacy, ultimately informing targeted interventions to improve digital health equity.

### Ethical Aspects Related to the Measurement of dHL

The act of measuring an individual’s dHL could potentially lead to shame and stigma among those with low literacy levels [8,10], highlighting the need for ethical considerations in the domain of dHL. Beyond this, ethical considerations should emphasize the importance of privacy, data security, and addressing potential disparities in access to digital health resources [40]. The development of tools that address digital competence and incorporate ethical dimensions ensures a comprehensive assessment framework that is sensitive to the ethical implications of digital health engagement.

Moreover, a global perspective is crucial when measuring dHL, given the variations in access to technology and health care infrastructure worldwide. Individuals with high dHL can effectively access health information through websites, health apps, and other digital platforms, depending on their location. However, those with limited dHL may struggle to navigate these online resources [7], increasing the risk of health inequalities. Adapting dHL assessment tools to accommodate these global variations ensures that assessments are culturally sensitive and applicable across diverse settings, contributing to a more equitable understanding of individuals’ dHL capabilities worldwide.

### Strengths and Limitations of the Method

To the author’s knowledge, this is the first comprehensive scoping review complemented by real-world insights from the Champions, providing a deeper understanding of the feasibility of dHL tools.

Scoping reviews often involve broad searches and numerous results, making them time-consuming. In line with scoping review methods, this mapping did not include a quality assessment of the studies, their evaluation and monitoring approaches, or whether the assessment tools used adequately measured HL. Studies incorporating HL measures were included based on the authors’ consideration that the intervention or program addressed aspects of HL and that the tools used served as measures of HL. However, the assessment of quality was derived from our network of Champions in a workshop setting.

The literature search covered a limited period (2018-2022), as the most commonly used assessment tools had already been validated before this time frame.

### Conclusion

dHL is a major determinant of health, yet its assessment is complex, influenced by factors at both individual and contextual levels. Although the eHEALS is the most frequently used tool, it has been criticized for its narrow scope, limited adaptability to the rapidly evolving digital landscape, and lack of objective testing.

There is a pressing need to develop future tools that not only reflect emerging trends and technologies but also encompass individual knowledge, skills, perceptions, and contextual factors.

It is worth noting that dHL is context specific and depends on health care systems and available technology, particularly the user interface. The primary objective of interventions targeting dHL should be to improve health rather than merely increase dHL levels in individuals and organizations. A comprehensive approach to dHL assessment tools—ensuring relevance and addressing diversity, including gender, socioeconomic status, and cultural factors—is crucial for a holistic understanding of dHL and its impact on health outcomes. Measuring dHL could also induce shame and stigma among those with low literacy levels, highlighting the need for ethical considerations, including privacy, data security, and addressing disparities in access to digital health resources (Textbox 3).

**Recommendations for choosing a digital health literacy assessment tool.**

- Consider a multidimensional tool that includes attitudes toward health and digital health, as well as trust in digital health services.
- Ensure the instrument is up-to-date, allowing it to capture the use of existing health technologies and digital services.
- Whenever possible, choose an evidence-based instrument, or consider validating the instrument yourself.
- Use an instrument that objectively evaluates functional skills.
- Let the purpose of your investigation guide the choice of the most appropriate instrument.

## Appendix

Multimedia Appendix 1 Search strategy for PubMed.

Multimedia Appendix 2 PRISMA-ScR (Preferred Reporting Items for Systematic reviews and Meta-Analyses extension for Scoping Reviews) checklist.

Multimedia Appendix 3 Detailed description of the papers included in the review.

## Abbreviations

dHL: digital health literacy
DHLI: Digital Health Literacy Instrument
eHEALS: eHealth Literacy Scale
eHL: eHealth literacy
eHLA: eHealth Literacy Assessment
eHLQ: eHealth Literacy Questionnaire
EU: European Union
HL: health literacy
HLS19-DIGI: the digital health literacy module of the Health Literacy Survey 2019
IDEAHL: Improving Digital Empowerment for Active and Healthy Living
TeHLI: Technology-Enabled Health Literacy Instrument
