# Supplementary material for: Choosing the Best Digital Health Literacy Measure for Research: Mixed Methods Study
Source: J Med Internet Res. 2025 Apr 8;27:e59807. doi: 10.2196/59807 (PMC12015337; doi:10.2196/59807)
Supplement: Multimedia Appendix 1 [file jmir_v27i1e59807_app1.pdf]

Appendix 1 Search strategy for PubMed

DOcumented **S**ystematic **I**nformation **S**earch

1. Describe your topic
**Task 1.2 (Electronic searches)**<br>**Identification of monitoring and evaluation frameworks and methods:**<br>• What monitoring and assessment tools, methods, and/or indicators exist for measuring HL and (d)HL in the EU and beyond (including national and regional variations)?<br>• How is the validation and sensitiveness documented in relation to these monitoring and assessment tools, methods, and/or indicators?<br>• What levels of HL and (d)HL are measured among the identified population groups in the EU and beyond?

2: Databases		
**Database name:**	**Platform**	**Date / search period**
**8: PubMed**		06.07.2022

3. Block indexing (selected search terms/search techniques for each block)				
**Database name:**	**Block 1:**<br>**Health Literacy /**<br>**Digital Health Literacy**	**Block 2:**<br>**European Union and Beyond**	**Block 3:**<br>**Assessment**	**Block 4:**<br>**Publication Type**
**8:**<br>**PubMed**	**Search terms/search techniques:**<br>• ("Computer*"[Title/Abstract] AND "Health"[Mesh] AND "Competenc*"[Title/Abstract])<br>• ("Computers"[Mesh] AND "Health"[Mesh] AND "Competenc*"[Title/Abstract])<br>• ("(d)HL"[Title/Abstract])<br>• ("d HL"[Title/Abstract])<br>• ("dHL"[Title/Abstract])	**Search terms/search techniques:**<br>• ("American*"[Title/Abstract])<br>• ("Australia"[Mesh])<br>• ("Australia"[Title/Abstract])<br>• ("Australia*"[Affiliation])<br>• ("Austria"[Affiliation])<br>• ("Austria"[Title/Abstract])<br>• ("Austrian*"[Title/Abstract])<br>• ("Belgian*"[Title/Abstract])<br>• ("Belgium"[Affiliation])<br>• ("Belgium"[Title/Abstract])	**Search terms/search techniques:**<br>• ("Assessment Framework"[Title/Abstract])<br>• ("Assessment Frameworks"[Title/Abstract])<br>• ("Assessments Framework"[Title/Abstract])<br>• ("Assessments Frameworks"[Title/Abstract])<br>• ("Assessment Method"[Title/Abstract])<br>• ("Assessment Methods"[Title/Abstract])<br>• ("Assessments Method"[Title/Abstract])<br>• ("Assessments Methods"[Title/Abstract])	**Search terms/search techniques:**<br>• ("Comment*"[Publication Type])<br>• ("Editorial*"[Publication Type])<br>• ("Letter*"[Publication Type])<br>• ("Study Protocol*"[Publication Type])

	• ("Digital Health Literacies"[Title/Abstract])<br>• ("Digital Health Literacy"[Title/Abstract])<br>• ("Digital Health Literate"[Title/Abstract])<br>• ("Digital Health"[Title/Abstract] AND "Competenc*"[Title/Abstract])<br>• ("Digital Literacies"[Title/Abstract])<br>• ("Digital Literacy"[Title/Abstract])<br>• ("Digital Literate"[Title/Abstract])<br>• ("eHealth Literacies"[Title/Abstract])<br>• ("e-Health Literacies"[Title/Abstract])<br>• ("eHealth Literacy"[Title/Abstract])<br>• ("e-Health Literacy"[Title/Abstract])<br>• ("eHealth Literate"[Title/Abstract])<br>• ("e-Health Literate"[Title/Abstract])<br>• ("eHealth"[Title/Abstract] AND "Competenc*"[Title/Abstract])<br>• ("e-Health"[Title/Abstract] AND "Competenc*"[Title/Abstract])<br>• ("Electronic Health Literacies"[Title/Abstract])	• ("Britain"[Title/Abstract])<br>• ("British"[Title/Abstract])<br>• ("Bulgaria"[Affiliation])<br>• ("Bulgaria"[Title/Abstract])<br>• ("Bulgarian*"[Title/Abstract])<br>• ("Canad*"[Title/Abstract])<br>• ("Canada"[Affiliation])<br>• ("Canada"[Mesh])<br>• ("Croatia"[Affiliation])<br>• ("Croatia"[Title/Abstract])<br>• ("Croatian*"[Title/Abstract])<br>• ("Cypriots"[Title/Abstract])<br>• ("Cyprus"[Affiliation])<br>• ("Cyprus"[Title/Abstract])<br>• ("Czech"[Affiliation])<br>• ("Czech*"[Title/Abstract])<br>• ("Danes"[Title/Abstract])<br>• ("Danish"[Title/Abstract])<br>• ("Denmark"[Affiliation])<br>• ("Denmark"[Title/Abstract])<br>• ("Dutch"[Title/Abstract])<br>• ("EEC"[Title/Abstract])<br>• ("England"[Affiliation])<br>• ("England"[Title/Abstract])<br>• ("English*"[Title/Abstract])<br>• ("Estonia"[Affiliation])<br>• ("Estonia"[Title/Abstract])<br>• ("Estonian*"[Title/Abstract])<br>• ("EU Countries*"[Title/Abstract])<br>• ("EU Country*"[Title/Abstract])<br>• ("EU Funded*"[Title/Abstract])<br>• ("Europe"[Mesh])	• ("Assessment Tool"[Title/Abstract])<br>• ("Assessment Tools"[Title/Abstract])<br>• ("Assessments Tool"[Title/Abstract])<br>• ("Assessments Tools"[Title/Abstract])<br>• ("Evaluation Framework"[Title/Abstract])<br>• ("Evaluation Frameworks"[Title/Abstract])<br>• ("Evaluations Framework"[Title/Abstract])<br>• ("Evaluations Frameworks"[Title/Abstract])<br>• ("Evaluation Method"[Title/Abstract])<br>• ("Evaluation Methods"[Title/Abstract])<br>• ("Evaluations Method"[Title/Abstract])<br>• ("Evaluations Methods"[Title/Abstract])<br>• ("Evaluation Scale"[Title/Abstract])<br>• ("Evaluation Scales"[Title/Abstract])<br>• ("Evaluations Scale"[Title/Abstract])<br>• ("Evaluations Scales"[Title/Abstract])<br>• ("Evaluation Score"[Title/Abstract])<br>• ("Evaluation Scores"[Title/Abstract])<br>• ("Evaluations Score"[Title/Abstract])<br>• ("Evaluations Scores"[Title/Abstract])<br>• ("Evaluation Test"[Title/Abstract])<br>• ("Evaluation Tests"[Title/Abstract])<br>• ("Evaluations Test"[Title/Abstract])<br>• ("Evaluations Tests"[Title/Abstract])<br>• ("Evaluation Tool"[Title/Abstract])<br>• ("Evaluation Tools"[Title/Abstract])<br>• ("Evaluations Tool"[Title/Abstract])	

	• ("Electronic Health Literacy"[Title/Abstract])<br>• ("Electronic Health Literate"[Title/Abstract])<br>• ("Electronic Health"[Title/Abstract] AND "Competenc*"[Title/Abstract])<br>• ("Health Information Literacies"[Title/Abstract])<br>• ("Health Information Literacy"[Title/Abstract])<br>• ("Health Information Literate"[Title/Abstract])<br>• ("Health Literacies"[Title/Abstract])<br>• ("Health Literacy"[Mesh])<br>• ("Health Literate"[Title/Abstract])<br>• ("Health"[Mesh] AND "Apps"[Title/Abstract] AND "Competenc*"[Title/Abstract])<br>• ("Health"[Mesh] AND "Digital Competenc*"[Title/Abstract])<br>• ("Internet"[Mesh] AND "Health"[Mesh] AND "Competenc*"[Title/Abstract])<br>• ("Internet"[Title/Abstract] AND "Health"[Mesh] AND "Competenc*"[Title/Abstract])	• ("Europe"[Title/Abstract])<br>• ("European Commission*"[Title/Abstract])<br>• ("European Common Market*"[Title/Abstract])<br>• ("European Communities"[Title/Abstract])<br>• ("European Community"[Title/Abstract])<br>• ("European Economic Communities"[Title/Abstract])<br>• ("European Economic Community"[Title/Abstract])<br>• ("European Parliament"[Title/Abstract])<br>• ("European Union"[Affiliation])<br>• ("European Union"[Mesh])<br>• ("European"[Title/Abstract])<br>• ("Finland"[Affiliation])<br>• ("Finland"[Title/Abstract])<br>• ("Finnish"[Title/Abstract])<br>• ("Finns"[Title/Abstract])<br>• ("France"[Affiliation])<br>• ("France"[Title/Abstract])<br>• ("French"[Title/Abstract])<br>• ("German*"[Title/Abstract])<br>• ("Germany"[Affiliation])<br>• ("Germany"[Title/Abstract])<br>• ("Great Britain"[Affiliation])<br>• ("Great Britain*"[Title/Abstract])<br>• ("Greece"[Affiliation])<br>• ("Greece"[Title/Abstract])<br>• ("Greek*"[Title/Abstract])<br>• ("Hungarian*"[Title/Abstract])	• ("Evaluations Tools"[Title/Abstract])<br>• ("Framework Assessment"[Title/Abstract])<br>• ("Framework Assessments"[Title/Abstract])<br>• ("Frameworks Assessment"[Title/Abstract])<br>• ("Frameworks Assessments"[Title/Abstract])<br>• ("Framework Evaluation"[Title/Abstract])<br>• ("Framework Evaluations"[Title/Abstract])<br>• ("Frameworks Evaluation"[Title/Abstract])<br>• ("Frameworks Evaluations"[Title/Abstract])<br>• ("Indicator"[Title/Abstract])<br>• ("Indicators"[Title/Abstract])<br>• ("Measurement"[Title/Abstract])<br>• ("Measurements"[Title/Abstract])<br>• ("Method Assessment"[Title/Abstract])<br>• ("Method Assessments"[Title/Abstract])<br>• ("Methods Assessment"[Title/Abstract])<br>• ("Methods Assessments"[Title/Abstract])<br>• ("Method Evaluation"[Title/Abstract])<br>• ("Method Evaluations"[Title/Abstract])<br>• ("Methods Evaluation"[Title/Abstract])<br>• ("Methods Evaluations"[Title/Abstract])<br>• ("Monitoring Test"[Title/Abstract])<br>• ("Monitoring Tests"[Title/Abstract])<br>• ("Monitorings Test"[Title/Abstract])<br>• ("Monitorings Tests"[Title/Abstract])<br>• ("Monitoring Tool"[Title/Abstract])<br>• ("Monitoring Tools"[Title/Abstract])	

	• ("Literacies Health"[Title/Abstract])<br>• ("Literacy Health"[Title/Abstract])<br>• ("Literate Health"[Title/Abstract])<br>• ("Media Health"[Title/Abstract] AND "Competenc*"[Title/Abstract])<br>• ("mHealth Literacies"[Title/Abstract])<br>• ("m-Health Literacies"[Title/Abstract])<br>• ("mHealth Literacy"[Title/Abstract])<br>• ("m-Health Literacy"[Title/Abstract])<br>• ("mHealth Literate"[Title/Abstract])<br>• ("m-Health Literate"[Title/Abstract])<br>• ("mHealth"[Title/Abstract] AND "Competenc*"[Title/Abstract])<br>• ("m-Health"[Title/Abstract] AND "Competenc*"[Title/Abstract])<br>• ("Mobile Health Literacies"[Title/Abstract])<br>• ("Mobile Health Literacy"[Title/Abstract])<br>• ("Mobile Health Literate"[Title/Abstract])<br>• ("Mobile Health"[Title/Abstract] AND "Competenc*"[Title/Abstract])	• ("Hungary"[Affiliation])<br>• ("Hungary"[Title/Abstract])<br>• ("Ireland"[Affiliation])<br>• ("Ireland"[Title/Abstract])<br>• ("Irish"[Title/Abstract])<br>• ("Italian*"[Title/Abstract])<br>• ("Italy"[Affiliation])<br>• ("Italy"[Title/Abstract])<br>• ("Latvia"[Affiliation])<br>• ("Latvia"[Title/Abstract])<br>• ("Latvian*"[Title/Abstract])<br>• ("Lithuania"[Affiliation])<br>• ("Lithuania"[Title/Abstract])<br>• ("Lithuanian*"[Title/Abstract])<br>• ("Luxembourg"[Affiliation])<br>• ("Luxembourg*"[Title/Abstract])<br>• ("Malta"[Affiliation])<br>• ("Malta"[Title/Abstract])<br>• ("Maltese"[Title/Abstract])<br>• ("Netherlands"[Affiliation])<br>• ("Netherlands"[Title/Abstract])<br>• ("New Zealand"[Affiliation])<br>• ("New Zealander"[Title/Abstract])<br>• ("Northern Ireland"[Affiliation])<br>• ("Northern Ireland"[Title/Abstract])<br>• ("Norway"[Affiliation])<br>• ("Norway"[Mesh])<br>• ("Norway"[Title/Abstract])<br>• ("Norwegian*"[Title/Abstract])<br>• ("Poland"[Affiliation])<br>• ("Poland"[Title/Abstract])<br>• ("Poles"[Title/Abstract])	• ("Monitorings Tool"[Title/Abstract])<br>• ("Monitorings Tools"[Title/Abstract])<br>• ("Reliability"[Title/Abstract])<br>• ("Reliable"[Title/Abstract])<br>• ("Scale Evaluation"[Title/Abstract])<br>• ("Scale Evaluations"[Title/Abstract])<br>• ("Scales Evaluation"[Title/Abstract])<br>• ("Scales Evaluations"[Title/Abstract])<br>• ("Score Evaluation"[Title/Abstract])<br>• ("Score Evaluations"[Title/Abstract])<br>• ("Scores Evaluation"[Title/Abstract])<br>• ("Scores Evaluations"[Title/Abstract])<br>• ("Screening Tool"[Title/Abstract])<br>• ("Screening Tools"[Title/Abstract])<br>• ("Screenings Tool"[Title/Abstract])<br>• ("Screenings Tools"[Title/Abstract])<br>• ("Sensitive"[Title/Abstract])<br>• ("Sensitivity"[Title/Abstract])<br>• ("Survey"[Title/Abstract])<br>• ("Surveys"[Title/Abstract])<br>• ("Test Evaluation"[Title/Abstract])<br>• ("Test Evaluations"[Title/Abstract])<br>• ("Tests Evaluation"[Title/Abstract])<br>• ("Tests Evaluations"[Title/Abstract])<br>• ("Test Monitoring"[Title/Abstract])<br>• ("Test Monitorings"[Title/Abstract])<br>• ("Tests Monitoring"[Title/Abstract])	

	• ("Online Health Literacies"[Title/Abstract])<br>• ("Online Health Literacy"[Title/Abstract])<br>• ("Online Health Literate"[Title/Abstract])<br>• ("Online*"[Title/Abstract] AND "Health"[Mesh] AND "Competenc*"[Title/Abstract])<br>• ("Smart Phone*"[Title/Abstract] AND "Health"[Mesh] AND "Competenc*"[Title/Abstract])<br>• ("Smartphone*"[Title/Abstract] AND "Health"[Mesh] AND "Competenc*"[Title/Abstract])<br>• ("Tablet*"[Title/Abstract] AND "Health"[Mesh] AND "Competenc*"[Title/Abstract])<br>• ("Telehealth"[Title/Abstract] AND "Health"[Mesh] AND "Competenc*"[Title/Abstract])<br>• ("Telemedicine"[Mesh] AND "Health"[Mesh] AND "Competenc*"[Title/Abstract])<br>• ("Telemedicine"[Title/Abstract] AND "Health"[Mesh] AND "Competenc*"[Title/Abstract])	• ("Polish"[Title/Abstract])<br>• ("Portugal"[Affiliation])<br>• ("Portugal"[Title/Abstract])<br>• ("Portuguese"[Title/Abstract])<br>• ("Romania"[Affiliation])<br>• ("Romania"[Title/Abstract])<br>• ("Romanian*"[Title/Abstract])<br>• ("Scotch"[Title/Abstract])<br>• ("Scotland"[Affiliation])<br>• ("Scotland"[Mesh])<br>• ("Scotland"[Title/Abstract])<br>• ("Scottish"[Title/Abstract])<br>• ("Slovakia"[Affiliation])<br>• ("Slovakia"[Title/Abstract])<br>• ("Slovaks"[Title/Abstract])<br>• ("Slovene*"[Title/Abstract])<br>• ("Slovenia"[Affiliation])<br>• ("Slovenia"[Title/Abstract])<br>• ("Spain"[Affiliation])<br>• ("Spain"[Title/Abstract])<br>• ("Spaniards"[Title/Abstract])<br>• ("Spanish"[Title/Abstract])<br>• ("Sweden"[Title/Abstract])<br>• ("Sweden"[Affiliation])<br>• ("Swedes"[Title/Abstract])<br>• ("Swedish"[Title/Abstract])<br>• ("UK"[Affiliation])<br>• ("United Kingdom"[Affiliation])<br>• ("United Kingdom"[Mesh])<br>• ("United Kingdom"[Title/Abstract])	• ("Tests Monitorings"[Title/Abstract])<br>• ("Tool Assessment"[Title/Abstract])<br>• ("Tool Assessments"[Title/Abstract])<br>• ("Tools Assessment"[Title/Abstract])<br>• ("Tools Assessments"[Title/Abstract])<br>• ("Tool Evaluation"[Title/Abstract])<br>• ("Tool Evaluations"[Title/Abstract])<br>• ("Tools Evaluation"[Title/Abstract])<br>• ("Tools Evaluations"[Title/Abstract])<br>• ("Tool Monitoring"[Title/Abstract])<br>• ("Tool Monitorings"[Title/Abstract])<br>• ("Tools Monitoring"[Title/Abstract])<br>• ("Tools Monitorings"[Title/Abstract])<br>• ("Tool Screening"[Title/Abstract])<br>• ("Tool Screenings"[Title/Abstract])<br>• ("Tools Screening"[Title/Abstract])<br>• ("Tools Screenings"[Title/Abstract])<br>• ("Validation Studies as Topic"[Mesh])<br>• ("Validation Study"[Publication Type])	

		• ("United States"[Affiliation])<br>• ("United States"[Mesh])<br>• ("United States"[Title/Abstract])<br>• ("Wales"[Affiliation])<br>• ("Wales"[Title/Abstract])<br>• ("Welsh"[Title/Abstract])		

**4. Search results for each individual block (number of hits)**				
**Database name:**	**Block 1: Health Literacy / Digital Health Literacy**	**Block 2: European Union and Beyond**	**Block 3: Assessment**	**Block 4: Publication Type**
**8: PubMed**	10.957	11.640.331	4.021.886	2.073.569

**4. Search results of various block combinations (number of hits)**			
**Database name:**	**Block 1 (Health Literacy / Digital Health Literacy) AND Block 2 (European Union and Beyond) AND Block 3 (Assessment)**	**Block 1 (Health Literacy / Digital Health Literacy) AND Block 2 (European Union and Beyond) AND Block 3 (Assessment) NOT Block 4 (Publication Type)**	**Block 1 (Health Literacy / Digital Health Literacy) AND Block 2 (European Union and Beyond) AND Block 3 (Assessment) NOT Block 4 (Publication Type) With limitations (part 5)**
**8: PubMed**	2.523	2.511	1.419

**5. Limitations (if any) due to scope of database (describe choices)**		
**Database name:**	**Year**	**Language**
**8: PubMed**	Filters: from 2018/1/1 - 3000/12/12	Filters: Danish, English, Finnish, French, German, Italian, Norwegian, Portuguese, Spanish, Swedish,

Appendix 2. Detailed description of the papers included in the review.

Author(s), year	Target group(s)	Tool(s)	(d)HL levels	Validation
**Austria**				
Maitz, E. et al. (2020) [41]	n=14 secondary school students aged 12–14.<br>All native German speakers and born in Austria or Germany.	eHEALS	eHEALS mean±SD score: 3.5±0.7 out of maximum 5.	Validation of the tool was not mentioned
**Cyprus**				
Efthymiou et al. 2019 [42]	n=101 Greek-speaking carers of people with dementia from Greece and Cyprus.<br><br>67% under 59 yr. old.<br>75.2% women.<br><br>53% with secondary and 39% tertiary education, 38% employed.<br>43% used internet to search for information.	eHeals-Carer	Results were not specified to Cypriot population alone.<br><br>Mean±SD eHeals-Carer score was 29.27±5.0 out of maximum 40 points. (Both Greek and Cypriot subjects included together).	The tool was validated in this article.<br><br>The tool had high internal consistency and high mean construct validity.
**Denmark**				
Bak et al. (2022) [43]	n=1518 university College students. 83.7% female.<br><br>Age mean±SD: 28.4 ± 8.4<br><br>49.5% studied education and 31% health education.<br><br>Subjective social status:<br>24% low,<br>62.7% medium,<br>8.6% high.	DHLI	59.9% sufficient,<br>**41.1% limited (d)HL.**<br><br>28.1% find it difficult to judge the quality and reliability of the information.	The tool has been validated elsewhere.

Author(s), year	Target group(s)	Tool(s)	(d)HL levels	Validation
Holt et al. (2020) [44]	n=366 nursing students. Aged 21–28 yr.92% female.<br><br>94% speak Danish at home.<br><br>71% with general upper secondary education.<br>33% with parents with medium education.<br>21% with chronic conditions.<br>57% with daily use of medication.	HLQ, eHLA	Mean HLQ scale scores (Q1–Q3) (entry-level students):<br>HLQ1: 2.96 (2.75–3.25)<br>HLQ2: 3.07 (3.00–3.25)<br>HLQ3: 2.80 (2.40–3.00)<br>HLQ4: 3.29 (3.00–3.80)<br>HLQ5: 2.83 (2.60–3.00)<br>HLQ6: 3.80 (3.40–4.20)<br>HLQ7: 3.70 (3.50–4.00)<br>HLQ8: 4.07 (3.80–4.20)<br>HLQ9: 3.97 (3.80–4.20)<br><br>Mean HLQ scale scores (Q1–Q3) (graduate-level students):<br>HLQ1: 2.93 (2.50–5.50)<br>HLQ2: 3.29 (3.00–3.75)<br>HLQ3: 2.95 (2.60–3.20)<br>HLQ4: 3.33 (3.00–3.80)<br>HLQ5: 3.02 (2.80–3.25)<br>HLQ6: 3.87 (3.60–4.20)<br>HLQ7: 3.84 (3.58–4.16)<br>HLQ8: 4.25 (4.00–4.60)<br>HLQ9: 4.18 (4.00–4.40)	The tools have been validated elsewhere.
Kayser et al. (2018) [45]	n=475 Danish Individuals from a wide range of settings.<br><br>Aged 16–74 yr.	eHLQ	Mean eHLQ scale scores (SD):<br><br>eHLQ1: 2.55 (0.66)<br>eHLQ2: 2.97 (0.55)<br>eHLQ3: 2.81 (0.69)<br>eHLQ4: 2.61 (0.66)<br>eHLQ5: 2.55 (0.65)<br>eHLQ6: 2.52 (0.55)<br>eHLQ7: 2.42 (0.62)	The tool has been validated elsewhere.
Karnoe et al. (2018) [46]	n=475 participants from the general Danish population.<br><br>30.9% aged 18–35 yr.,<br>36.6% aged 36–60 yr.,<br>28% aged 60+ yr.<br><br>51.6% female,<br>47.2% with long education. 41.3% with excellent self-rated health, 39.8% with chronic conditions.	eHLA	No dHL levels were reported in this validation article.	The tool was validated in this article.<br><br>The eHLA provides the means for gaining insight into people’s health-related literacy as well as their confidence, familiarity, and motivation related to digital solutions. This toolkit consists of 7 tools that validly measure constructs with a satisfactory fit to log linear RMs, thus displaying essential validity and objectivity.
Holt et al. (2019) [47]	n=246 Danish adult outpatients	eHLQ, eHLA	Mean eHLQ scale scores (1–4):<br>eHLQ1: 2.7<br>eHLQ2: 3.1	The tool has been validated elsewhere.

Author(s), year	Target group(s)	Tool(s)	(d)HL levels	Validation
	from Gentofte Hospital.<br><br>55.7% female.<br><br>26.4% with long education. 43.9% with well health. 37.4% with diabetes and 62.6% with other condition.		eHLQ3: 3.0<br>eHLQ4: 2.8<br>eHLQ5: 2.7<br>eHLQ6: 2.7<br>eHLQ7: 2.6<br><br>Mean eHLA scale scores:<br>Functional HL: 9.5<br>Self-assessed HL: 3.3<br>Familiarity with health and disease: 3.1<br>Knowledge of health and disease: 9.7<br>Digital familiarity: 3.5<br>Digital confidence: 3.4<br>Digital incentives: 3.5	
Kayser et al. (2022) [48]	n=194 members of a Danish medical staff (mean age 34). Mean age 43.1 yr.<br><br>85.1% female.	eHLQ	Mean eHLQ scale scores (SD):<br>eHLQ1: 2.98 (0.60)<br>eHLQ2: 3.40 (0.47)<br>eHLQ3: 3.36 (0.51)<br>eHLQ4: 2.95 (0.42)<br>eHLQ5: 2.78 (0.45)<br>eHLQ6: 2.57 (0.40)<br>eHLQ7: 2.55 (0.51)	The tool was validated in this article. Construction and validity testing in a broad range of target groups generated clear evidence of construct validity, discriminant validity, and scale reliability. This initial validity testing indicates that the eHLQ is likely to be valuable for the characterization and understanding
**Germany**				
Dadaczynski et al. (2022a) [49]	n=490 German grade 8–9 students. Subjective social status medium 64.3%. Physical activity 3 or more days a week 67.3%. Fruit consumption daily 42.4%	DHLI	**24.6%** (dimension scores varying from 15.3% to 37.5%) **of adolescents reported difficulties in acquiring and dealing with digital health information**.<br><br>Stratified by social characteristics, gender and socioeconomic differences were found with girls and respondents reporting a lower SSS more often showed a limited (d)HL.	Validation of the tool was not mentioned.
Mayer (2018) [50]	n=100, 18–35-yr.-old university students from Germany.<br>First yr. students 18%, 20% second yr., 27% third yr. and 35 fourth yr. and above. 5 participants had	EHILS, HLS-EU-Q47	HLS-EU-Q47: total mean score 2.75 (SD=0.32).<br>EHILS: total mean score 3,15 (SD=0.94)	The validity of EHILS in German was tested in this article. A positive correlation (r= .47) was found between the EHILS10 and the HLS-EU-Q47 total score. Internal consistency of the EHILS was low, corroborating that health information literacy is a heterogeneous construct. Regarding validity, differential correlations of the overall EHILS scores as well as the

Author(s), year	Target group(s)	Tool(s)	(d)HL levels	Validation
	been involved in professional or voluntary work in the medical sector.			subindices motivation and confidence with HL measures, domain-specific self-efficacy beliefs, generalized internal control beliefs, and health information searching experiences were found. It is concluded that ability and motivation components of EHILS should be assessed separately to understand individuals’ health information behaviour.
Dadaczynski et al. (2021) [51]	n=14 916 university students (mean age 23.3)	Five aspects of DHLI adapted to context of COVID-19 pandemic	Across (d)HL dimensions, the greatest difficulties could be found for assessing the reliability of health-related information (42.3%) and the ability to determine whether the information was written with a commercial interest (38.9%). Moreover, the respondents indicated that they most frequently have problems finding the information they are looking for (30.4%)<br>Female university students reported a lower DHLI for the dimensions of "information searching" and "evaluating reliability"	Validation of the tool was not mentioned.
De Santis et al. (2021) [52]	n=1014 participants 14 to 93 yr. (mean age 54) from Munich, Germany.<br><br>66% with tertiary education, 60% were either employed or seeking employment. 45% with net household income of up to 3500€. 57.1% used digital technology for health purposes.	eHEALS	eHEALS mean score: 31 out of maximum 40.<br><br>A higher perceived eHL score was associated with younger age, higher household income, and more education.	The tool has been validated elsewhere.

Author(s), year	Target group(s)	Tool(s)	(d)HL levels	Validation
Marsall et al. (2022) [53]	n=470 German speaking adults aged 18–82 yr. (mean age 37.1).<br><br>51.9% from big cities. 58.1% had university degree. 61.3% had internet always available.	eHEALS	eHEALS mean score 31.2 out of maximum 40.<br><br>Information seeking: Mean 3.85 (SD=0,86).<br>Information appraisal: Mean 3.95 (SD=0,74).	The tool was validated in this article. The newly revised GR-eHEALS questionnaire represents a valid instrument to measure the important health-related construct eHL. GR-eHEALS has high content validity, good internal consistency and reliability.
Pfob et al. (2021) [54]	n=113 individuals, of which 61.9% IT specialists and 38.1% health care specialist.	eHLA	A high or the highest level of HL was reported by 23.9% of all survey participants which, analysed by profession, corresponds to 0.0% of the IT specialists and 62.8% of the healthcare specialists.<br>In general, health care specialists scored significantly higher on the four health-related scales, whereas IT specialists scored significantly higher on the three digitally related scales.	The tool has been validated elsewhere.
Knitza et al. (2020) [55]	n=193 German adults with musculoskeletal and rheumatic diseases (mean age 52).<br><br>91% regularly used a mobile phone. 38 % lived in villages, 25% in small cities, 18 % in mid-sized cities, 18 % in big cities.	eHEALS	eHEALS mean score: 26.3 (SD 7.1) out of maximum 40 (Women: 25.8, men: 27.0)<br>Age showed a negative correlation with eHEALS score.	The tool has been validated elsewhere.
Heiman et al. (2018) [56]	n=182 German patients with cancer and their caregivers (mean age 50.7).	eHEALS	Summarizing the five questions used, the mean score was 14.7, with a score range from 5 to 25.<br><br>58.5% of the patients had a score above the average,<br><br>41.5% had a low score for eHL.	The tool has been validated elsewhere.

Author(s), year	Target group(s)	Tool(s)	(d)HL levels	Validation
Atmann et al. (2021) [57]	n=129 Asthma patients from Germany (mean age 55).<br><br>94% with school diploma, 52% employed. 62% mild, 29% moderate and 9% severe asthma.	HLS-EU-Q16, eHEALS	HLS-EU-Q16:<br>47% sufficient,<br>32% problematic,<br>**21% inadequate HL.**<br><br>Mean score of eHEALS dimensions:<br>3.1 out of maximum 5.<br>No consistent differences between trained and untrained groups were found, suggesting that trained patients did not benefit from asthma education regarding HL and eHL.	The tools have been validated elsewhere.
**Greece**				
Trantali et al. (2022) [58]	n=113 health sciences students –undergraduate health sciences students at Greek Universities in Greece (19.6% in Attica region), of 18 yr. of age or older (mean=22; min=18, max=53).<br><br>Most of them were women (81.4%), were not working (n=82, 72.6%), were single (n=65, 57.5%) and live with their parents (n=60, 53.1%). Recruitment via Facebook, survey in Google Forms.	eHEALS	eHEALS score mean: 31.9 out of maximum 40.<br><br>Medicine and dentistry students had the highest score (33.7) and other health and caring sciences students the lowest (29.8).<br><br>There was a statistically significant difference at eHEALS score among University Departments (p=0.009).	The tool has been validated elsewhere
Efthymiou et al. (2019) [42]	n=101 carers of people with dementia. 75.2% women, 67.3% aged less than 60 yr.<br><br>53% had secondary education. 38 % were employed.	eHEALS-Carer	eHEALS-carer mean score: 29.27 out of maximum 40.	The tool was validated in this article (reliability and validity):<br>- High internal consistency (Cronbach's alpha): .083.<br>- High Mean I-CVI (0.93) (Construct validity)<br>Content validation was also assessed by an expert panel of 10 professionals.

Author(s), year	Target group(s)	Tool(s)	(d)HL levels	Validation
	43% used the internet to search for information. In addition, an expert panel of 10 was invited for content validation of the tool.			
Kritsotakis et al. (2021) [59]	n=200 staff nurses (60.5%) and nursing assistants (39.5%) from Greece. 91% were female and 35% were between 45 and 54 yr. old.<br><br>66% had middle-level financial status and 49.5% had high education level. 79% with leadership status.	eHEALS	eHEALS mean score: 30.7 out of maximum 40.<br><br>The lowest mean value (SD) was 3.24 (1.07) (range: 1–5), for the confidence in using information from the Internet and the highest (SD) was 4.08 (0.76) on how to find helpful health resources on the Internet.	The tool has been validated elsewhere
**Hungary**				
Zrubka et al. (2019) [60]	n=1000 respondents (mean age: 46.3 yr., range: 18–90) from the Middle (34.8%), East (35.3%) and West (29.9%) of Hungary.<br>55% female, 34.6% had obtained secondary education and 29.6% had higher education.	Hungarian eHEALS	eHEALS mean score: 29.2 out of maximum 40.<br><br>Small, but statistically significant differences of eHEALS scores between males and females, as well as older (>65) and younger adults, but no differences between individuals with low education or low income and the rest of the sample.	eHEALS (HU): The tool was validated in this article. Internal consistency was good (Cronbach's α = 0.90), and test–retest reliability was moderate (intraclass correlation r = 0.64). The Hungarian eHEALS is a useful and valid tool for measuring subjective eHL.
Zrubka et al. (2020) [61]	n=666 respondents recruited online from the Hungarian general population, 18–	eHEALS	eHEALS mean score 29.3 out of maximum 40.<br><br>eHL is associated with patient-reported experiences.	The tool has been validated elsewhere.

Author(s), year	Target group(s)	Tool(s)	(d)HL levels	Validation
	65 + yr. (mean: 48.9; SD: 17.6).<br><br>Respondents with tertiary education and from the highest income quintile were slightly over-represented, whereas rural citizens were slightly under-represented compared with the general population.			
**Ireland**				
Delemere et al. (2021) [62]	n=85 participants, of which 57 were parents of children with cancer and n=28 were their Health Care Providers	eHEALS	eHL:<br>All (mean (SD): 30.80 (7.25); Parents 29.98 (6.37); Health Care Providers 32.48 (8.68).<br>In conclusion, this article has highlighted the importance of eHL and device use on Connected Health for Health Care Providers and parents of children with cancer.	The tool has been validated elsewhere.
**Italy**				
Lorini, et al. (2022a) [63]	n=3025 university students, mean age 23.1 yr. (SD 5.0),<br>All had access to the internet in the previous four weeks to answering the survey to search for information regarding COVID-19.	COVID-19 Digital HL Instrument (COVID-19 DHLI)	No total HL levels were reported in this validation article.	Cronbach alpha values are acceptable for all DHLI subscales (ranging from 0.74 to 0.83) except for the privacy subscale (0.39), indicating reliability for all but privacy. 4 of 5 DHLI subscales' response distribution covered all response options adequately with no floor or ceiling effects, showing that the instrument is good enough to assess the variability of the phenomenon. Construct validity, as revealed by correlation analyses, appears adequate.

Author(s), year	Target group(s)	Tool(s)	(d)HL levels	Validation
Del Giudice et al. (2018) [64]	n=868 Italians aged 20–30 yr. recruited from University of Udine, student mailing lists and Facebook contacts of the research team members. Educational attainment high in 44.1%. 45.1% employed, 47.1% articleing. Self-rated health very bad in 0.7%, poor in 7.1%. 12.0% used internet for health purposes several times a week.	eHEALS	The total mean score of Italian eHEALS in the whole population was 28.2 out of maximum 40.<br>Real-life working or articleing experiences in the health sector, as a proxy of higher levels of HL, positively correlate with self-referred eHL as measured by the eHEALS.	The tool was validated in this article. Psychometric properties were examined by measuring internal consistency (Cronbach alpha) and conducting a principal component analysis to assess the dimensionality of the scale. The scale shows good internal consistency and construct validity.
Bevilacqua et al. (2021) [65]	n=58 older adults, mean age of 68.2 yr., primary education 8.6%, secondary education 70.7%, tertiary education 20.7%.	eHEALS	eHEALS mean was 24.3 out of maximum 40 at baseline and 28.4 after the intervention.	The tool has been validated elsewhere.
**Poland**				
Burzynska et al. 2022 [66]	n=1527 social media users (mean age 32) from Poland. 89.8% female. 75.2% university graduates. 60.3% good or very good health.	eHEALS	The mean total score of eHEALS-Pl for the evaluated population was found to be 30.69 ± 4.25. 31 or less points indicate low score<br>49.2% respondents obtained a high and<br>**50.8% a low eHEALS-Pl score.**	The reliability of the eHEALS-Pl was measured by calculating the Cronbach alpha coefficients and analysing the principal components. Exploratory factor analysis and hypothesis testing was used to assess the construct validity of the instrument. The internal consistency of the eHEALS-Pl was sufficient.
Duplaga et al 2020 [67]	n=1030, mean age (SD) of the respondent 26.09 (4.87) yr., 100% female. 41.7% of inhabitants of rural areas. Married 40.0%.	HLS-EU-Q16, eHEALS	The mean HL score (HLS-EU-Q16) was 11.87<br><br>53.3% sufficient,<br>20.8% problematic,<br>**20.9 % inadequate HL.**<br>eHL score 29.52 out of maximum 40.	The tools have been validated elsewhere.

Author(s), year	Target group(s)	Tool(s)	(d)HL levels	Validation
	With children 60.4%			
**Portugal**				
Martins et al. (2022) [68]	n=1815 university students (mean age 24,2). 87.9% Portuguese.<br>75.1% females.<br>Most studied bachelor's degree (51%). 36.5% were enrolled in social sciences and 35.2% in Health sciences studies.<br>90.8% had subjective social status Median and above.	DHLI adapted to the COVID-19 pandemic	Not reported.	The tool was validated in this article. The article aimed to translate, adapt and validate the Portuguese version of the dHL Instrument as used in the global COVID-HL Network. The Portuguese version of the DHLI met adequate psychometric criteria. Therefore, it can be confidently used in Portuguese students' assessment of dHL. Representative studies are needed to shed light on different target groups and their COVID-19–related DHLI.
Arriaga et al. (2022) [69]	n=1247 people from mainland Portuguese population (mean age 46).<br>92.1% were born in Portugal, 2.2% in Brazil and 1.8% in Angola.<br>41.1% reported good and 32.2% fair health regarding self-health perception.<br>64.7% reported not having a long-term illness or health problems.<br>70.8% considered that health problems did not limit their usual activities.	HLS19-Q12	6.1% excellent,<br>41.2% sufficient,<br>25.0% problematic,<br>**27.7% inadequate Digital HL.**	The tool was validated in this article.<br><br>This article aimed to describe the process of adaptation to Portugal of the short-form version of the HL Survey (HLS19-Q12) from the HL Population Survey Project 2019–2021, also establishing the HL levels in the Portuguese population.<br><br>The overall data suggest the HLS19-Q12 as a feasible measure to assess HL in the Portuguese population. Thus, it can be used in Portugal to assess the population’s needs and monitor and evaluate policies and initiatives to promote HL by addressing its societal, environmental, personal, and situational modifiable determinant factors.
**Slovenia**				
Vrdelja et al. (2021) [70]	n=3621 male and female university students, mean age 22.6 yr.	DHLI (3 subscales, adapted for Covid-19,	85.4% did not have problems assessing usefulness of information. 82.4% did not have problems using information in everyday life.	Validation of the tool was not mentioned.

Author(s), year	Target group(s)	Tool(s)	(d)HL levels	Validation
	21.0% had high socioeconomic status, 14.3% low status	Slovenian translation)	86.4% could use information to make decisions about their own health. Students with sufficient (d)HL more often sought information from official institutions.<br><br>**27.9% had difficulties in finding useful information. 29.6% had problems choosing among information sources found. 49.3% had difficulties assessing the reliability of information**. Students with limited (d)HL more often sought information	
**Sweden**				
Wångdahl et al. (2020) [71]	n=348 adults from Sweden (mean age 49 yr.). 90.4% with at least 10 yr. of education. 85.8% perceived their own general health as good or very good. 87.9% used internet almost every day.	HLS-EU-Q16, eHEALS	HLS-EU-Q16:<br>71.5% sufficient,<br>**22% problematic,**<br>**6% inadequate** comprehensive HL.<br><br>The mean sum score of Sw-eHEALS (Swedish version of eHEALS) was 29.3, referring to a **sufficient level**.	The Swedish version of eHEALS was validated in this article.<br>eHEALS was assessed as being unidimensional with high internal consistency of the instrument, making the reliability adequate.
Bergman et al. (2021) [72]	n=681 Arabic speaking migrants (n=344) and Swedish speaking residents (n=337) (mean age 45.9 yr.).<br><br>49.8% graduated from university.<br><br>77.1% good or very good self-perceived health.<br><br>85.9% used internet almost every day.	HLS-EU-Q16, eHEALS	HLS-EU-Q16:<br>55.5% sufficient,<br>**31.2% problematic,**<br>**13.3% inadequate** comprehensive HL.<br><br>eHEALS:<br>67.5% sufficient,<br>**24.8% problematic,**<br>**7.7% inadequate** dHL.<br><br>Arabic speakers had significantly lower mean sum scores in eHL 28.1 (SD 6.1) vs 29.3 (6.2) and **lower proportion of sufficient CHL 125 (38.9%) vs 239 (71.3%)**, compared to Swedish speakers.	The tools have been validated elsewhere.

Author(s), year	Target group(s)	Tool(s)	(d)HL levels	Validation
Wångdahl et al. (2021) [73]	n=298 Arabic speaking adults from Sweden (mean age 41 yr.). Mean±SD 9,4±8.2 yr. lived in Sweden. 53% graduated from university. 67.7% good or very good self-perceived health. 85.9% used internet every day.	HLS-EU-Q16, Ar-eHEALS	HLS-EU-Q16: 38.4% sufficient, **39.4% problematic,** **22.1% inadequate** HL. Ar-eHEALS: 62.2% sufficient, **28.7% problematic,** **8.9% inadequate** HL. Mean ± SD 28,1 ± 6,1. Range: 8-40.	The Ar-eHEALS tool was validated in this article. The psychometric testing showed that the Ar-eHEALS is valid and reliable and can be used to assess eHL among Arabic speaking people in Sweden.

[1] ‘World Health Organization. The global burden of chronic diseases, Global Burden of Disease (GBD) (healthdata.org) (Assessed 20th February 2024).’.

[2] ‘World Economic Forum. Our health systems are under pressure. Here are 9 ways to remedy that (Assessed the 20th of February 2024)’.

[3] J. R. McColl-Kennedy *et al.*, ‘The changing role of the health care customer: review, synthesis and research agenda’, *Journal of Service Management*, vol. 28, no. 1, pp. 2–33, 2017, doi: 10.1108/JOSM-01-2016-0018.

[4] ‘Canadian Public Health Association. Low health Literacy and Chronic Disease prevention and Control- Perspectives from The Health And Public Health Sectors. 2006’.

[5] K. Sørensen *et al.*, ‘Health literacy and public health: A systematic review and integration of definitions and models’, 2012. doi: 10.1186/1471-2458-12-80.

[6] ‘Health Promotion Glossary of Terms 2021’.

[7] ‘World Health Organization (2023) Digital health literacy key to overcoming barriers for health workers, WHO study says’.

[8] I. Kickbusch, J. M. Pelikan, F. Apfel, A. D. Tsouros, and World Health Organization. Regional Office for Europe, *Health literacy : the solid facts*.

[9] J. N. Haun, M. A. Valerio, L. A. McCormack, K. Sørensen, and M. K. Paasche-Orlow, ‘Health literacy measurement: An inventory and descriptive summary of 51 instruments’, Oct. 25, 2014, *Bellwether Publishing, Ltd.* doi: 10.1080/10810730.2014.936571.

[10] A. Pleasant, ‘Advancing health literacy measurement: A pathway to better health and health system performance’, *J Health Commun*, vol. 19, no. 12, pp. 1481–1496, Dec. 2014, doi: 10.1080/10810730.2014.954083.

[11] The IDEAHL consortium, ‘IDEAHL – Improving Digital Empowerment for Active Healthy Living’. Accessed: Oct. 23, 2023. [Online]. Available: https://ideahl.eu/

[12] Z. Munn *et al.*, ‘What are scoping reviews? Providing a formal definition of scoping reviews as a type of evidence synthesis’, Apr. 04, 2022, *Lippincott Williams and Wilkins*. doi: 10.11124/JBIES-21-00483.

[13] ‘Global Health Literacy Acadent Health Literacy ATLAS’.

[14] Health Literacy Europe, ‘Health Literacy Eurpope’, Accessed: Feb. 07, 2023. [Online]. Available: https://www.healthliteracyeurope.net/

[15] ‘EuroHealthNet’.

[16] ‘eHealth Action Plan 2012-2020 ’.

[17] ‘Horizon 2020 ’.

[18] ‘IC-Health ’.

[19] ‘Digital Health Europe’.

[20] ‘Health Literacy in the Nordic Countries ’.

[21] ‘DHE´s practice catalogue ’.

[22] ‘European Health Literacy Survey’.

[23] ‘Health Literacy Tool Shed (bu.edu) ’.

[24] ‘The HLS-EU questionnaire ’.

[25] ‘The M-POHL network action ’.

[26] ‘WHO Health Literacy Road Map’.

[27] ‘Covidence’, Covidence org. Accessed: Jan. 24, 2023. [Online]. Available: https://www.covidence.org/

[28] ‘IDEAHL’, EU. Accessed: Feb. 07, 2023. [Online]. Available: https://ideahl.eu/

[29] D. Pollock *et al.*, ‘Moving from consultation to co-creation with knowledge users in scoping reviews: Guidance from the JBI Scoping Review Methodology Group’, *JBI Evid Synth*, vol. 20, no. 4, pp. 969–979, Apr. 2022, doi: 10.11124/JBIES-21-00416.

[30] S. Elo and H. Kyngäs, ‘The qualitative content analysis process’, *J Adv Nurs*, vol. 62, no. 1, pp. 107–115, Apr. 2008, doi: 10.1111/j.1365-2648.2007.04569.x.

[31] ‘D1.1. Report on (d)HL WP1’. Accessed: Feb. 20, 2024. [Online]. Available: https://cordis.europa.eu/project/id/101057477/results

[32] A. Faux-Nightingale, F. Philp, D. Chadwick, B. Singh, and A. Pandyan, ‘Available tools to evaluate digital health literacy and engagement with eHealth resources: A scoping review’, Aug. 01, 2022, *Elsevier Ltd*. doi: 10.1016/j.heliyon.2022.e10380.

[33] C. Délétroz, M. C. Allen, M. Sasseville, A. Rouquette, P. Bodenmann, and M. P. Gagnon, ‘eHealth literacy measurement tools: a systematic review protocol’, *Syst Rev*, vol. 11, no. 1, Dec. 2022, doi: 10.1186/s13643-022-02076-2.

[34] J. Lee, E. H. Lee, and D. Chae, ‘eHealth literacy instruments: Systematic review of measurement properties’, Nov. 01, 2021, *JMIR Publications Inc.* doi: 10.2196/30644.

[35] A. Karnoe Lars Kayser, A. Karnoe, and L. Kayser, ‘Knowledge Management & E-Learning How is eHealth literacy measured and what do the measurements tell us? A systematic review How is eHealth literacy measured and what do the measurements tell us? A systematic review’, *Knowledge Management & E-Learning*, vol. 7, no. 4, pp. 576–600, 2015.

[36] T. H. Nguyen, M. K. Paasche-Orlow, and L. A. Mccormack, ‘The State of the Science of Health Literacy Measurement HHS Public Access’, 2017. [Online]. Available: www.healthliteracybu.edu

[37] L. Xie and P. K. H. Mo, ‘Comparison of eHealth Literacy Scale (eHEALS) and Digital Health Literacy Instrument (DHLI) in Assessing Electronic Health Literacy in Chinese Older Adults: A Mixed-Methods Approach’, *Int J Environ Res Public Health*, vol. 20, no. 4, Feb. 2023, doi: 10.3390/ijerph20043293.

[38] J. K. Fujioka *et al.*, ‘Challenges and Strategies for Promoting Health Equity in Virtual Care: Protocol for a Scoping Review of Reviews’, Dec. 01, 2020, *JMIR Publications Inc.* doi: 10.2196/22847.

[39] B. Crocker, O. Feng, and L. R. Duncan, ‘Performance-based Measurement of eHealth Literacy: A Systematic Scoping Review (Preprint)’, *J Med Internet Res*, Jun. 2022, doi: 10.2196/44602.

[40] ‘GDPR’.

[41] E. Maitz *et al.*, ‘Internet-based health information-seeking behavior of students aged 12 to 14 years: Mixed methods study’, *J Med Internet Res*, vol. 22, no. 5, May 2020, doi: 10.2196/16281.

[42] A. Efthymiou, N. Middleton, A. Charalambous, and E. Papastavrou, ‘Adapting the eHealth Literacy Scale for Carers of People With Chronic Diseases (eHeals-Carer) in a Sample of Greek and Cypriot Carers of People With Dementia: Reliability and Validation Study’, *J Med Internet Res*, vol. 21, no. 11, p. e12504, 2019, doi: 10.2196/12504.

[43] C. K. Bak *et al.*, ‘Digital Health Literacy and Information-Seeking Behavior among University College Students during the COVID-19 Pandemic: A Cross-Sectional Study from Denmark’, *Int J Environ Res Public Health*, vol. 19, no. 6, 2022, doi: 10.3390/ijerph19063676.

[44] K. A. Holt, D. Overgaard, L. V Engel, and L. Kayser, ‘Health literacy, digital literacy and eHealth literacy in Danish nursing students at entry and graduate level: a cross sectional study’, *BMC Nurs*, vol. 19, p. 22, 2020, doi: 10.1186/s12912-020-00418-w.

[45] L. Kayser *et al.*, ‘A Multidimensional Tool Based on the eHealth Literacy Framework: Development and Initial Validity Testing of the eHealth Literacy Questionnaire (eHLQ)’, *J Med Internet Res*, vol. 20, no. 2, p. e36, 2018, doi: 10.2196/jmir.8371.

[46] A. Karnoe, D. Furstrand, K. B. Christensen, O. Norgaard, and L. Kayser, ‘Assessing Competencies Needed to Engage With Digital Health Services: Development of the eHealth Literacy Assessment Toolkit’, *J Med Internet Res*, vol. 20, no. 5, p. e178, 2018, doi: 10.2196/jmir.8347.

[47] K. A. Holt *et al.*, ‘Differences in the Level of Electronic Health Literacy Between Users and Nonusers of Digital Health Services: An Exploratory Survey of a Group of Medical Outpatients’, *Interact J Med Res*, vol. 8, no. 2, p. e8423, 2019, doi: 10.2196/ijmr.8423.

[48] L. Kayser *et al.*, ‘Health Professionals’ eHealth Literacy and System Experience Before and 3 Months After the Implementation of an Electronic Health Record System: Longitudinal Study’, *JMIR Hum Factors*, vol. 9, no. 2, p. e29780, 2022, doi: 10.2196/29780.

[49] K. Dadaczynski *et al.*, ‘[Digital health literacy of pupils. Level and associations with physical activity and dietary behavior]’, *Bundesgesundheitsblatt Gesundheitsforschung Gesundheitsschutz*, vol. 65, no. 7–8, pp. 784–794, 2022, doi: 10.1007/s00103-022-03548-5.

[50] A. K. Mayer, ‘Examining the factorial structure and validity of the everyday health information literacy screening tool’, *Cogent Med*, vol. 5, no. 1, pp. 1–14, 2018, doi: 10.1080/2331205X.2018.1545378.

[51] K. Dadaczynski *et al.*, ‘Digital Health Literacy and Web-Based Information-Seeking Behaviors of University Students in Germany During the COVID-19 Pandemic: Cross-sectional Survey Study’, *J Med Internet Res*, vol. 23, no. 1, p. e24097, 2021, doi: 10.2196/24097.

[52] K. K. De Santis, T. Jahnel, E. Sina, J. Wienert, and H. Zeeb, ‘Digitization and Health in Germany: Cross-sectional Nationwide Survey’, *JMIR Public Health Surveill*, vol. 7, no. 11, p. e32951, 2021, doi: 10.2196/32951.

[53] M. Marsall, G. Engelmann, E. M. Skoda, M. Teufel, and A. Bäuerle, ‘Measuring Electronic Health Literacy: Development, Validation, and Test of Measurement Invariance of a Revised German Version of the eHealth Literacy Scale’, *J Med Internet Res*, vol. 24, no. 2, p. e28252, 2022, doi: 10.2196/28252.

[54] A. Pfob *et al.*, ‘Contrast of Digital and Health Literacy Between IT and Health Care Specialists Highlights the Importance of Multidisciplinary Teams for Digital Health-A Pilot Study’, *JCO Clin Cancer Inform*, vol. 5, pp. 734–745, 2021, doi: 10.1200/CCI.21.00032.

[55] J. Knitza *et al.*, ‘Mobile Health Usage, Preferences, Barriers, and eHealth Literacy in Rheumatology: Patient Survey Study’, *JMIR Mhealth Uhealth*, vol. 8, no. 8, p. e19661, 2020, doi: 10.2196/19661.

[56] H. Heiman, C. Keinki, and J. Huebner, ‘EHealth literacy in patients with cancer and their usage of web-based information’, *J Cancer Res Clin Oncol*, vol. 144, no. 9, pp. 1843–1850, 2018, doi: 10.1007/s00432-018-2703-8.

[57] O. Atmann, C. Werner, K. Linde, and A. Schneider, ‘Health literacy and eHealth among adult asthma patients - results of a cross sectional survey’, *J Asthma*, vol. 58, no. 2, pp. 262–270, 2021, doi: 10.1080/02770903.2019.1672720.

[58] T. Trantali, C. Athanasopoulou, A. Lagiou, and E. Sakellari, ‘eHealth Literacy Among Health Sciences Students in Greece’, *Stud Health Technol Inform*, vol. 289, pp. 252–255, 2022, doi: 10.3233/SHTI210907.

[59] G. Kritsotakis, E. Andreadaki, M. Linardakis, G. Manomenidis, T. Bellali, and P. Kostagiolas, ‘Nurses’ ehealth literacy and associations with the nursing practice environment’, *Int Nurs Rev*, vol. 68, no. 3, pp. 365–371, 2021, doi: 10.1111/inr.12650.

[60] Z. Zrubka, O. Hajdu, F. Rencz, P. Baji, L. Gulácsi, and M. Péntek, ‘Psychometric properties of the Hungarian version of the eHealth Literacy Scale’, *Eur J Health Econ*, vol. 20, pp. 57–69, 2019, doi: 10.1007/s10198-019-01062-1.

[61] Z. Zrubka *et al.*, ‘Exploring eHealth Literacy and Patient-Reported Experiences With Outpatient Care in the Hungarian General Adult Population: Cross-Sectional Study’, *J Med Internet Res*, vol. 22, no. 8, p. e19013, 2020, doi: 10.2196/19013.

[62] E. Delemere, R. Maguire, I. S. S. U. G. I. Technol, and W. Univ, ‘Technology usage, eHealth literacy and attitude towards connected health in caregivers of paediatric cancer’, *IEEE International Symposium on Technology and Society (ISTAS) - Technological Stewardship and Responsible Innovation*, 2021, doi: 10.1109/istas52410.2021.9629210.

[63] C. Lorini *et al.*, ‘Validation of the COVID-19 Digital Health Literacy Instrument in the Italian Language: A Cross-Sectional Study of Italian University Students’, *Int J Environ Res Public Health*, vol. 19, no. 10, 2022, doi: 10.3390/ijerph19106247.

[64] P. Del Giudice *et al.*, ‘Correlation Between eHealth Literacy and Health Literacy Using the eHealth Literacy Scale and Real-Life Experiences in the Health Sector as a Proxy Measure of Functional Health Literacy: Cross-Sectional Web-Based Survey’, *J Med Internet Res*, vol. 20, no. 10, p. e281, 2018, doi: 10.2196/jmir.9401.

[65] R. Bevilacqua *et al.*, ‘eHealth Literacy: From Theory to Clinical Application for Digital Health Improvement. Results from the ACCESS Training Experience’, *Int J Environ Res Public Health*, vol. 18, no. 22, 2021, doi: 10.3390/ijerph182211800.

[66] J. Burzyńska, M. Rękas, and P. Januszewicz, ‘Evaluating the Psychometric Properties of the eHealth Literacy Scale (eHEALS) among Polish Social Media Users’, *Int J Environ Res Public Health*, vol. 19, no. 7, 2022, doi: 10.3390/ijerph19074067.

[67] M. Duplaga, ‘The Use of Fitness Influencers’ Websites by Young Adult Women: A Cross-Sectional Study’, *Int J Environ Res Public Health*, vol. 17, no. 17, 2020, doi: 10.3390/ijerph17176360.

[68] S. Martins *et al.*, ‘Adaptation and validation and of the Digital Health Literacy Instrument for Portuguese university students’, *Health Promot J Austr*, 2022, doi: 10.1002/hpja.580.

[69] M. Arriaga *et al.*, ‘Health Literacy in Portugal: Results of the Health Literacy Population Survey Project 2019-2021’, *Int J Environ Res Public Health*, vol. 19, no. 7, 2022, doi: 10.3390/ijerph19074225.

[70] M. Vrdelja, S. Vrbovšek, V. Klopčič, K. Dadaczynski, and O. Okan, ‘Facing the Growing COVID-19 Infodemic: Digital Health Literacy and Information-Seeking Behaviour of University Students in Slovenia’, *Int J Environ Res Public Health*, vol. 18, no. 16, 2021, doi: 10.3390/ijerph18168507.

[71] J. Wångdahl, M. Jaensson, K. Dahlberg, and U. Nilsson, ‘The Swedish Version of the Electronic Health Literacy Scale: Prospective Psychometric Evaluation Study Including Thresholds Levels’, *JMIR Mhealth Uhealth*, vol. 8, no. 2, p. e16316, 2020, doi: 10.2196/16316.

[72] L. Bergman, U. Nilsson, K. Dahlberg, M. Jaensson, and J. Wångdahl, ‘Health literacy and e-health literacy among Arabic-speaking migrants in Sweden: a cross-sectional study’, *BMC Public Health*, vol. 21, no. 1, p. 2165, 2021, doi: 10.1186/s12889-021-12187-5.

[73] J. Wångdahl, K. Dahlberg, M. Jaensson, and U. Nilsson, ‘Arabic Version of the Electronic Health Literacy Scale in Arabic-Speaking Individuals in Sweden: Prospective Psychometric Evaluation Study’, *J Med Internet Res*, vol. 23, no. 3, p. e24466, 2021, doi: 10.2196/24466.
